# Other Gynecologic Cancers: endometrial, ovarian, vulvar and vaginal cancers

**DOI:** 10.1186/1472-6874-4-S1-S14

**Published:** 2004-08-25

**Authors:** Eliane Duarte-Franco, Eduardo L Franco

**Affiliations:** 1 Departments of Oncology and Family Medicine, McGill University, Montreal, Canada; 2 Departments of Oncology and Epidemiology and Biostatistics, McGill University, Montreal, Canada

## Abstract

**Health issue:**

In Canada, cancers of the endometrium, ovaries, vulva, vagina, placenta and adnexa account for 11% of all malignant neoplasms in women and 81% of all genital cancers. Although the incidence and mortality from vulvar and vaginal cancers are very low, endometrium and ovarian cancer are important public health problems.

**Key findings:**

In Canada, there has been no appreciable improvement in survival for women with advanced endometrial (EC) or ovarian cancer (OC) over the past 30 years. The prognosis of EC is good for most patients because diagnosis is made at early stages. However, survival of OC is poor; more than 70% of cases are diagnosed at late stages. Up to 10% of OCs is linked to familial aggregation. Cancers of the vulva and of the vagina are very rare. The survival experience for women with the latter is worse than for those with the former. Both share many risk factors with cervical cancer and the recent developments in the study of HPV infection should be applicable to these diseases as well. Of particular interest will be the advent of vaccines for the primary prevention of HPV infection.

**Data gaps and recommendations:**

At present, the best available means to diagnose gynecologic malignancies is a detailed clinical examination, considering the totality of information on potential and proven risk factors, such as age, reproductive health, sexual practices, use unopposed estrogens or of oral contraceptives or tubal ligation, obesity, diet, smoking, and the familial clustering of some of these cancers.

## Background

In 2000, there were more than 4.7 million cases of cancer in women worldwide, 54% of which occurred in less developed countries. [1] Gynecologic tumours, including cancer of the endometrium, ovary, vulva, vagina, placenta and adnexa, accounted for 8% of all female primary tumours worldwide and 45% of all genital cancers (cervical cancer is discussed in the chapter entitled "Cancer of the Uterine Cervix"). In Canada, these diseases make up 11% of all neoplasias in women and 81% of all genital cancers.

Although the incidence and mortality from endometrial and ovarian cancer are important public health problems, other female genital cancers are very rare. Primary tumours of the vagina, vulva, placenta and adnexa total 0.6% of all female cancers. Neoplasia developing elsewhere in the female genital tract is even less common. Because neoplastic disease in these sites is rare, there is very little information on it; most of the data available are from case series or hospital- and clinic-based studies.

## Methods

Data on incidence rates and rates of mortality from gynecologic cancers in Canada were obtained from Statistics Canada and in the United States from the Surveillance, Epidemiology, and End Results (SEER) Program. Other epidemiologic and risk factor information was found in published surveillance and research reports.

## Results

### Cancer of the Endometrium

#### Incidence and Mortality

Most neoplasias of the corpus of the uterus develop in the endometrium and are endometrioid type adenocarcinomas. Endometrial cancer (EC) is the seventh most common malignancy affecting women worldwide, with almost 190,000 cases annually.[2] It is the most common uterine malignancy in Canada, accounting for 43% of all female genital cancers. [1]

**Figure 1:**
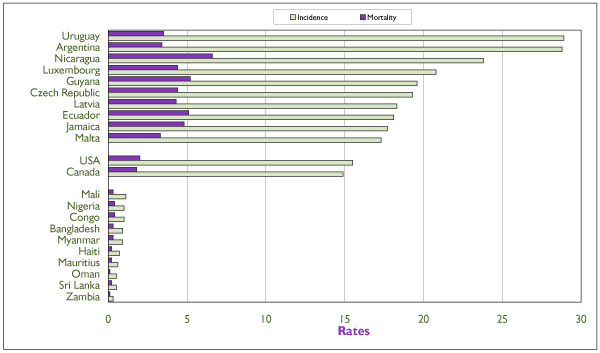
**Annual age-standardized incidence and mortality rates of invasive endometrial cancer (projection for 2000) for the 10 highest-risk and the 10 lowest-risk countries (based on incidence), for Canada and for the United States. **Rates are per 100,000 and are standardized according to the world population of 1960. Source: GLOBOCAN, International Agency for Research on Cancer. [1]

There is great variation in the incidence rates of EC internationally. The highest risk areas are Central and North America and Polynesia, whereas rates that are 10 times lower occur in western Africa. Figure 1 shows incidence and mortality rates for the 10 countries with the highest and the 10 with the lowest incidence rates, and for Canada and the United States. Incidence rates in the latter two countries are among the 20 highest worldwide (14.9 and 15.5 per 100,000 respectively). High-risk countries with better resources tend to have relatively low mortality rates.

**Figure 2:**
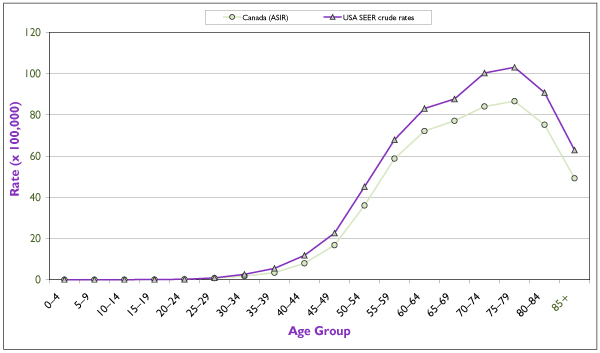
**Average (period 1994–1998) age-specific incidence rates for invasive endometrial cancer in Canada and in the United States. **Incidence data for the United States refer to the 11 main state or metropolitan area registries belonging to the Surveillance, Epidemiology, and End Results (SEER) Program from the Cancer Surveillance Research Program of the U.S. National Cancer Institute (NCI). Source: Statistics Canada, SEER database. [4]

**Figure 3:**
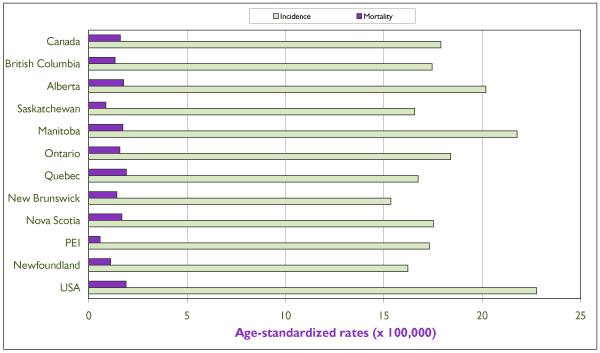
**Average (period 1994–1998) annual age-standardized incidence and mortality rates of invasive endometrial cancer for individual Canadian provinces, for Canada and for the United States. **All rates refer to numbers of new cases or deaths per 100,000 and are standardized according to the Canadian population of 1991. Source: Statistics Canada and SEER database. [4]

An estimated 3,500 new cases were diagnosed in Canada in 2001, which is more than twice the number of cervical cancer cases. [3] The age-specific incidence curves for Canada and the United States are rather similar (Figure 2), the differences in incidence being reflected somewhat proportionally across all age groups. This suggests similar distributions of risk factors in the female populations of the two countries. The incidence of invasive endometrial cancer rises sharply after the age of 40 and begins to decline after 75 to 79 years of age. The variation in annual incidence rates among Canadian provinces is relatively small, from 15.1/100,000 in New Brunswick to 21.5/100,000 in Manitoba (Figure 3).

#### Time Trends

**Figure 4:**
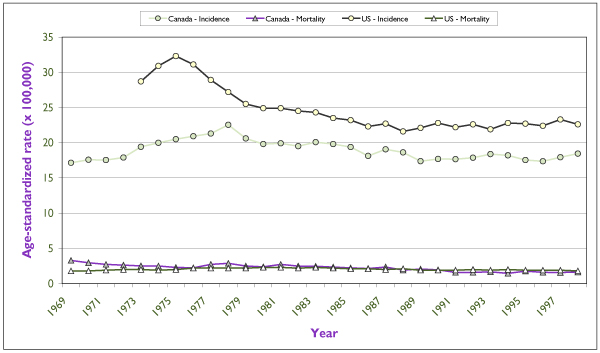
**Trends in incidence and mortality rates for invasive endometrial cancer for Canada and for the United States in the last 30 years. **Rates are per 100,000 and are standardized according to the Canadian population of 1991. U.S. incidence data refer to the nine main SEER registries, and mortality data refer to the entire U.S. population. Source: Statistics Canada and SEER database. [4]

Figure 4 shows the trends in annual incidence and mortality rates for EC in Canada and in the United States. Rates were steadily increasing until the mid-1970s, most likely as a reflection of the widespread use in North America of unopposed estrogens as hormone therapy for post-menopausal symptoms. As soon as the etiologic association was recognized, use of unopposed estrogens declined dramatically, and incidence rates also began to decline. This effect seems to have been delayed by about four years in Canada. Since the late 1980s incidence rates have levelled off or have begun to increase slightly. In general, mortality rates have not accompanied the variations in incidence to any important degree. They have remained low, at around 2/100,000. The trends within Canadian provinces are similar.

#### Survival

The prognosis of EC is generally favourable because in 75% to 90% of cases the disease is confined to the uterus at diagnosis, a condition that is highly curable by surgical removal of the uterus. [5] The overall five-year survival rate is 75%. [65] In addition to stage at diagnosis, survival from EC is also greatly influenced by histological type and grade of the tumour (the higher the stage, the poorer the prognosis). The five-year survival rates are as follows: 88% for stage I, 73% for stage II, 52% for stage III, and 27% for stage IV. [7] Over the past 30 years there has been no appreciable improvement in survival among women with advanced disease. In the United States there are striking differences in survival according to race: over the last three decades, Black women have faced an overall survival rate 30% to 40% lower than that of white women. [6] Stage at diagnosis, hormonal and reproductive characteristics, socio-economic level and access to health care explain 80% of this difference. [8]

#### Risk Factors

EC risk is influenced by reproductive and hormonal factors. The greatest impact on the risk of endometrial cancer comes from the exposure to estrogens without concomitant intake of progesterone. Several case-control studies have demonstrated the effects of estrogen replacement therapy on the risk of EC. [9] In premenopausal women sequential oral contraceptives (OCs), which were largely used in the past, led to substantial increases in risk, whereas modern combination OCs generally decrease risk by approximately 50%. Maximum protection may be conferred to women who receive low-dose estrogen and high-dose progestin. [10] Tamoxifen, a non-steroidal drug with both estrogen and anti-estrogen effects, used largely to treat breast cancer and to prevent the occurrence of a second breast cancer, improves survival and reduces the risk of second breast primaries but increases the risk of endometrial cancers. [11]

Nulliparity, [12,13] high socio-economic status, [14] multiple births, increased maternal age at first delivery [15] and diabetes [16] confer an increased risk of EC. Unlike the case with many cancers, EC riskis lower among women who smoke. [17,18] The risk factor profiles for invasive and pre-invasive endometrial cancers have been found to be similar. [19]

Obesity has consistently been shown to be associated with increased risk (two to five times as great) of EC, possibly through a combination of mechanisms. Obese women may be more prone to an increased endogenous production of estradiol. Increased risk may also result from continued anovulation, causing progesterone deficiency. Several studies have examined the relation between EC and physical activity because exercise lowers serum estrone levels. Most studies report a weak to moderate protective effect, but this question is still unsettled, since the effect does not vary with the duration and intensity level of activity and it is difficult to determine whether obesity, a strong risk factor, is a cause or a consequence of inactivity. [16]

Diets rich in whole grains, fresh fruit and vegetables as well as consumption of fatty fish exert a protective effect against EC, whereas high animal fat intake seems to increase EC risk. [16,20,21] A recent Canadian study found a reduced risk of EC with high consumption of vegetables, fibre, vitamin E and alcohol. [22]

#### Screening Strategies

Women receiving unopposed estrogens should undergo endometrial sampling biennially (risk increases only after two years of estrogen use), particularly if endometrial hyperstimulation has previously been documented and has not been treated by short-term administration of progestins. At present, there are no guidelines for the surveillance of women using tamoxifen, and in particular those taking tamoxifen who have used estrogen replacement therapy in the past. Because these women may be at increased risk of endometrial cancer, information on abnormal bleeding should be obtained on a routine basis; decisions on additional tests should be taken by their gynecologists. Use of newer estrogen antagonists, such as raloxifene, which seem to provide similar preventive effects for breast cancer and none of the risk effects for endometrial cancer, are likely to change this perception. [23]

Women at high risk who request an endometrial evaluation may undergo an office-based investigative procedure to rule out pathology. [24] An endometrial evaluation should also be performed in women with a history of Lynch II syndrome, a condition associated with a very high risk of endometrial carcinoma.[25]

High-risk women should be evaluated by any of the following detection tests: (i) cervico-vaginal cytology, (ii) endometrial cytology, (iii) endometrial biopsy, (iv) dilatation and curettage, (v) transvaginal ultrasonography, (vi) magnetic resonance imaging or computerized tomography, or (vii) hysteroscopy.

The only group of women for whom there are explicit guidelines for screening are those with a positive test or positive family history of hereditary non-polyposis colon cancer. The American Cancer Society recommends annual screening beginning at age 35. [26]

Routine screening of asymptomatic women using any of the techniques noted has not been proven to be of benefit. [27] No screening test has been assessed with respect to its impact on mortality from endometrial cancer. The Pap test lacks sufficient sensitivity to reliably detect endometrial cancer or its precursors, but it may help to reveal the presence of endometrial cells on a smear of a post-menopausal woman not taking exogenous hormones. [28,29]

One could argue that although EC is common, its attendant morbidity is relatively low as compared with that of other female neoplasms, such as breast and cervical cancers. The high cost and discomfort to the patient of most procedures make them unattractive as large-scale, cost-effective screening tools.

#### Primary Prevention

Preventing EC through public and professional education seems to be a sensible approach. Physician education should include increasing awareness that EC is the most common gynecologic malignancy and that risk can be reduced by early intervention with endometrial biopsy and treatment in the setting of abnormal uterine bleeding, by administration of progestational therapy for post-menopausal patients on estrogen-only regimens, and by referral for endometrial biopsy of those patients who have endometrial or atypical glandular cells on Pap smear. Public education concerning the risks of endometrial cancer should include awareness of (i) the need to maintain a proper weight, (ii) the risks associated with unopposed estrogen therapy, (iii) the value of OC use in reducing risk, and (iv) the most common signs and symptoms of the disease. [24]

### Cancer of the Ovary

#### Incidence and Mortality

Accounting for 3% of all cancers in women and figureing more than 165,000 new cases estimated in2001, ovarian cancer is the sixth most frequent female cancer and the sixth leading cause of death in women worldwide. [2] In Canada, where ovarian cancer accounts for 4% of all female cancers or 31%ofall genital cancers in women, it is estimated that in 2001 there were 2,500 new cases and 1,500 deaths.[3]

**Figure 5:**
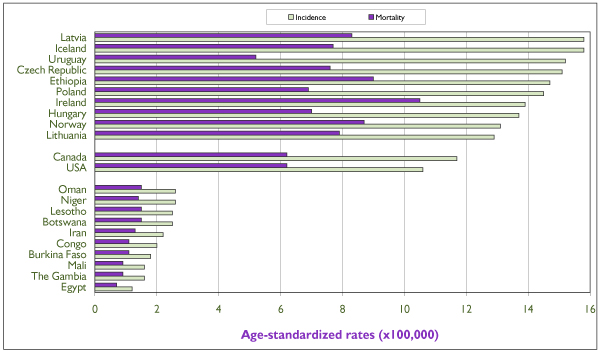
**Annual age-standardized incidence and mortality rates of invasive ovarian cancer (projection for 2000) for the 10 highest-risk and the 10 lowest-risk countries (based on incidence), for Canada and for the United States. **Rates are per 100,000 and are standardized according to the world population of 1960. Source: GLOBOCAN, International Agency for Research on Cancer. [1]

There is substantial variation in incidence rates of ovarian cancer internationally, from 3/100,000 in central Africa to 13/100,000 in northern Europe. Developing nations have incidence rates that are approximately half those of the most resource-rich countries. Canada and the United States are among the countries with the highest rates of ovarian cancer worldwide, with rates of 12 and 11 per 100,000 respectively [1] (Figure 5). Within countries, mortality rates are generally not good indicators of the region's incidence, and unlike the situation for other gynecologic cancers, mortality from ovarian cancer does not seem to be related to the country's level of development.

**Figure 6:**
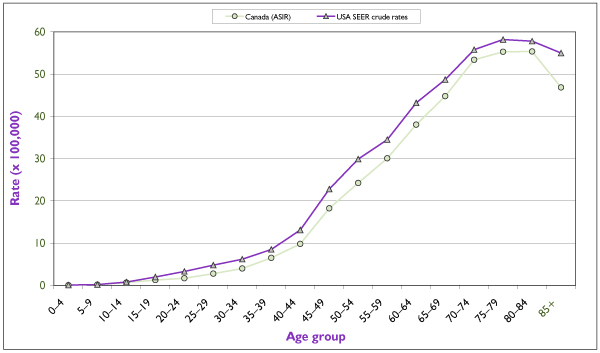
**Average (period 1994–1998) age-specific incidence rates for invasive ovarian cancer in Canada and in the United States. **U.S. incidence data refer to the 11 main SEER registries. Source : Statistics Canada, SEER database. [4]

**Figure 7:**
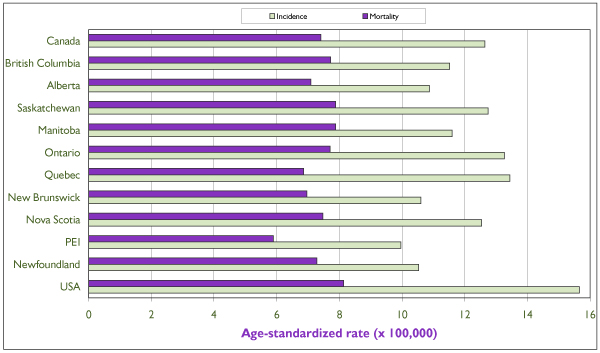
**Average (period 1994–98) annual age-standardized incidence and mortality rates of invasive ovarian cancer for individual Canadian provinces, for Canada and for the United States. **All rates refer to numbers of new cases or deaths per 100,000 and are standardized according to the Canadian population of 1991. Source: Statistics Canada and SEER database. [4]

The incidence of ovarian cancer rises sharply after the age of 40 and peaks in the eighth decade of life [3,4] (Figure 6). In Canada, 63% of cases are diagnosed after the age of 50. [30] Canadian incidence and mortality rates show little variation. The lowest age-standardized incidence rate is 10/100,000 in Prince Edward Island, and the highest is 13.7/100,000 in Quebec [31] (Figure 7).

**Figure 8:**
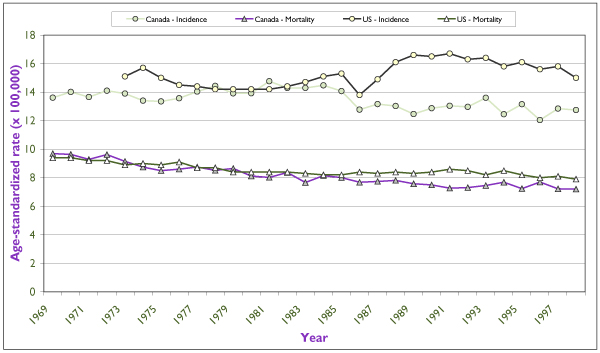
**Trends in incidence and mortality rates for invasive ovarian cancer for Canada and for the United States in the last 30 years**. Rates are per 100,000 and are standardized according to the Canadian population of 1991. U.S. incidence data refer to the nine main SEER registries, and mortality data refers to the entire U.S. population. Source: Statistics Canada and SEER database. [4]

Figure 8 shows time trends in the incidence and mortality of ovarian cancer in Canada and in the United States for the past 30 years. The rates in Canada have been fairly stable in the past 15 years. In the United States the marked and abrupt increase in incidence in the late 1980s probably reflects more active case finding. Mortality rates in Canada have declined slowly over the past 30 years but seem to have levelled off in recent years. Substantial reductions in incidence between 1969 and 1998 were reported in British Columbia, Manitoba and Quebec (data not shown).

Most ovarian cancers are epithelial in origin, but there are many different histological presentations of the tumour. Nonetheless, there seems to be evidence for the existence of precancerous lesions, [32] although these are difficult to study because of their location and because the disease is usually detected only in advanced stages, only 25% being diagnosed while still localized. [30]

#### Survival

Ovarian cancer tends to be a fatal disease because it is usually not detected before extensive intraperitoneal spread. The most important factor in predicting survival from ovarian cancer is stage at diagnosis. [33] Since 70% of patients present with stage III or IV at diagnosis, the five-year survival rate for all stages of ovarian cancer is less than 40%, although women with stage I disease have five-year survival rates between 80% and 90%. [34] Women with disease confined to the ovaries are four times more likely to survive for five years than women who have a diagnosis of distant metastatic disease.

#### Risk Factors

Of all ovarian cancers, 5% to 10% are hereditary, and most of these (65% to 75%) belong to the hereditary breast-ovarian cancer syndrome group that is linked to mutations in BRCA1 or BRCA2 tumour suppressor genes. [30,35] The median age at diagnosis of familial ovarian cancer is considerably lower, at approximately 59 years, than that for sporadic cancers. [36]

Some reproductive factors result in significant protection against ovarian cancer: OC use reduces the risk by 30% to 40% and so does a term pregnancy (40%). [10,37,38] Tubal ligation has been associated with a reduction in risk of 60% and hysterectomy with 30%. [37]. Both OC use and tubal ligation have also shown substantial protection against ovarian cancer in BRCA1 and BRCA2 mutation carriers. [39,40]

Several other risk factors (e.g. diet, alcohol) that have been studied have not shown consistent effects.

#### Screening Strategies

Several factors render screening for ovarian cancer impractical: there is no defined precursor lesion as the target, there are many possible etiologic pathways, access to the ovaries is difficult, and most ovarian pathologies that produce an abdominal mass are not malignant.

Tumour markers, of which the CA 125 test is the most promising, have been the focus of considerable attention. The U.S. NCI-PDQ^® ^program has evaluated the CA 125 test, transvaginal ultrasound, and pelvic examination and has concluded that there is insufficient evidence to recommend any of these methods. Similar conclusions were reached by the U.S. Preventive Services Task Force and by the Canadian Task Force on Preventive Health Care. [41] Large randomized trials are currently under way that use a multi-modal approach testing for the serum tumour marker CA 125 followed by transvaginal ultrasound, but preliminary results are still conflicting. Additional data are needed to fully evaluate the impact of screening on morbidity, mortality, health economics and psychosocial issues. [42]

#### Primary Prevention

OC use, tubal ligation and prophylactic oophorectomy have been suggested as possible manoeuvres to prevent ovarian cancer in women who have a strong family history or have been found to be positive for a BRCA mutation. However, none of these approaches has been shown to reduce the mortality from ovarian cancer or the incidence of advanced disease.

### Cancer of the Vulva

#### Incidence and Mortality

Although tumours in the vulva occur two to three times as frequently as those in the vagina, they are still a rare finding. The cancer registry reporting the most elevated incidence rate of vulvar cancer is that of Prince Edward Island in Canada (2.6/100,000 women). Rates above 2/100,000 are found elsewhere, in Peru and Brazil (2.5/100,000 and 2.3/100,000 respectively). [43] All provinces in Canada have rates above 1/100,000 women; these are among the highest reported worldwide.

#### Survival

The overall survival rate of vulvar cancer is 46%. [44] The main prognostic feature in cancer of the vulva is lymph node involvement. Stage I disease has a five-year survival rate ranging from 90% to 94%, stage II from 81% to 91%, stage III from 36% to 74%, and stage IV from 19% to 31%. [45]

#### Risk Factors

There seem to be two distinct risk factor profiles for vulvar cancer that are age-dependent. Cancers developing in younger women (< 65 years) tend to have the same risk profile as other anogenital cancers, i.e. association with low socio-economic status, high-risk sexual behaviours, and the presence of human papillomavirus (HPV) infection and smoking. [46-48] In addition, these neoplasms occur more frequently in women with a diagnosis of other cancers, particularly those related to HIV infection and cigarette smoking. [47] Risk of vulvar cancer and of its precursor, vulvar intraepithelial neoplasia (VIN), is substantially greater among immunocompromised women; in fact, 1% to 37% of HIV-positive women also have VIN. [48]

Women with late-onset vulvar cancers (55 to 85 years) are typically without a previous history of sexually transmitted infections and tend not to be smokers. HPV DNA is found in only 15% of these cancers. [47]

It is not yet well understood whether the trend of increasing rates of vulvar neoplasia is due to greater public and physician awareness about the risk of HPV-associated diseases or whether it is a cohort effect associated with changes in sexual practices that began in the late 1960s, such as increased sexual activity and increased prevalence of smoking among women of reproductive age.

#### Prevention

Because there is evidence of diagnostic delays, that is, women seek medical care in advanced stages of the disease, some authors recommend that women perform self-examination monthly and also that physicians be made aware of the features of this disease. [48] Given that smoking is an important risk factor of vulvar as well as of other primary anogenital cancers, patients who smoke should be advised and encouraged to quit. [49] Women with HIV infection should be monitored for anogenital lesions in the vulva, vagina and cervix.

### Cancer of the Vagina

#### Incidence and Mortality

Neoplasms of the vagina are extremely rare. The reported annual age-standardized incidence rate (ASIR) is fewer than 1/100,000 women in all countries with the exception of Argentina (1.5/100,000) and Colombia (1.2/100,000). [43] Nova Scotia is the Canadian province with the highest ASIR for vaginal cancers, at 0.8/100,000, ranked fourth highest in the world. For the other reporting provinces, rates vary between 0.3/100,000 and 0.7/100,000. Since neoplasia found in the vagina may represent metastasis or infiltration of primary cancers occurring elsewhere in the body, it is possible that such rates are inflated.

#### Survival

The survival of patients with vaginal cancers is generally poor: stage I disease has a five-year survival rate of 64% to 90%, stage II of 29% to 66%, and stages III and IV of 0% to 40%. [44]

#### Risk Factors

Most vaginal cancers are squamous. Clear cell adenocarcinomas used to occur in young women as a consequence of maternal exposure to diethylstilbestrol (DES) before the 12th week of gestation. [50] Other risk factors include hysterectomy, endometriosis, and frequent vaginitis due to chronic irritation. [50,51] More recently, an association between exposure to DES and increased risk of squamous neoplasia of the cervix and the vagina was found in long-term follow-up of two large cohorts of exposed women who were compared with unexposed controls. [52]

A recent population-based study found that HPV infection, lifetime number of sexual partners, early age at first intercourse, and smoking were associated with an increased risk of in situ and invasive vaginalneoplasia.[53] In addition, a higher risk was associated with a history of other anogenital cancers and previous hysterectomy (in the absence of previous anogenital cancer).[53] HPV DNA is found in themajority of vaginal tumours, pointing to a role for the virus in the etiology of vaginal cancers, as has been demonstrated for cervical cancer.[53,54] Because most women infected with HPV do not developcancer, it is possible that genetic susceptibility plays an important part. Daling et al.[53] found that women with a first-degree relative with anogenital cancer had almost three times the risk of vaginal cancer.

#### Prevention

Because cancer in the vagina may appear in association with other anogenital cancers, the follow-up of women with a diagnosis of these other cancers should include routine cytological screening and colposcopic examination of the vagina. The health care provider should keep in mind that this procedure should be performed even if the patient has had a hysterectomy.

Smoking cessation should be advised, since smoking is a risk factor for cancer of the vagina as well as for neoplasms of other anogenital sites. In addition, the risk of a second cancer for women with cancer of the vagina and of the vulva is greater than that expected according to the incidence rate of these cancers. A study using data from the U.S. SEER program found that most of the excess second cancers were related to smoking (lung, esophagus and upper respiratory tract) or to HPV infection (cervix, vulva, vagina and anus). [49]

## Discussion

### Data Limitations

Although there is a relative wealth of information on the factors that may cause women to be at higher risk of any of these gynecologic cancers, data on the efficacy of various screening strategies are still incomplete

## Recommendations

Modifiable risk factors such as exposure to unopposed estrogens, obesity and diet play an important role in increasing the risk of endometrial cancer. Therefore, public and professional education to prevent this disease should be encouraged and improved. Concerning risk factors, there is good evidence for a protective role of oral contraceptives, which should be taken into consideration by practitioners when judging risks of familial ovarian cancer.

Although several screening methods have been studied, including several tumour markers, none has been found yet to be particularly useful in reducing the incidence of advanced disease or mortality from ovarian cancer; thus further research is needed to develop diagnostic methods capable of diagnosing disease at an early stage. At present, the best available means of diagnosing gynecologic malignancies is through a detailed clinical examination as part of routine health care, provided that the physician considers the totality of information on potential and proven risk factors, such as age, reproductive health, sexual practices, smoking and the familial clustering of some of these cancers.
